# (*R*)-(−)-Aloesaponol III 8-Methyl Ether from *Eremurus persicus*: A Novel Compound against Leishmaniosis

**DOI:** 10.3390/molecules22040519

**Published:** 2017-03-24

**Authors:** Daniela Rossi, Karzan Mahmood Ahmed, Raffaella Gaggeri, Serena Della Volpe, Lauretta Maggi, Giuseppe Mazzeo, Giovanna Longhi, Sergio Abbate, Federica Corana, Emanuela Martino, Marisa Machado, Raquel Varandas, Maria do Céu Sousa, Simona Collina

**Affiliations:** 1 Department of Drug Sciences, Medicinal Chemistry and Pharmaceutical Technology Section, University of Pavia, Viale Taramelli 12, 27100 Pavia, Italy; daniela.rossi@unipv.it (D.R.); karzchem@yahoo.com (K.M.A.); raffaella.gaggeri@irst.emr.it (R.G.); serena.dellavolpe01@universitadipavia.it (S.D.V.); lauretta.maggi@unipv.it (L.M.); 2 Department of Science-Chemistry, University of Garmian, Kalar 46021, Kurdistan Region, Iraq; 3 Istituto Scientifico Romagnolo per lo Studio e la Cura dei Tumori (IRST) Srl—IRCCS Via Piero Maroncelli, 40, 47014 Meldola (FC), Italy; 4 Dipartimento di Medicina Molecolare e Traslazionale, Università di Brescia, Viale Europa 11, 25123 Brescia, Italy; giuseppe.mazzeo@unibs.it (G.M.); giovanna.longhi@unibs.it (G.L.); smrmachado@gmail.com (S.A.); 5 Centro Grandi Strumenti, University of Pavia, Via Bassi 21, 27100 Pavia, Italy; federica.corana@unipv.it; 6 Department of Earth and Environmental Sciences, University of Pavia, Via S. Epifanio 14, 27100 Pavia, Italy; 7 CESPU, Instituto de Investigação e Formação Avançada em Ciências e Tecnologias da Saúde, 4585-116 Gandra PRD, Portugal; smrmachado@gmail.com; 8 CIBIO-UP, Centro de Investigação em Biodiversidade e Recursos Genéticos, Universidade do Porto, InBIO, 4485-661 Vairão, Portugal; 9 Faculty of Pharmacy, University of Coimbra, Pólo das Ciências da Saúde, Azinhaga de Santa Comba, 3000-548 Coimbra, Portugal; raquel.varandas@ci.uc.pt (R.V.); mcsousa@ci.uc.pt (M.d.C.S.); 10 CNC—Center for Neurosciences and Cell Biology, University of Coimbra, Rua Larga Faculty of Medicine, Pólo I, 3004-504 Coimbra, Portugal

**Keywords:** leishmaniosis, drug identification, *Eremurus persicus*, plant extract, (*R*)-aloesaponol III-8 methyl ether

## Abstract

Leishmaniosis is a neglected tropical disease which affects several millions of people worldwide. The current drug therapies are expensive and often lack efficacy, mainly due to the development of parasite resistance. Hence, there is an urgent need for new drugs effective against *Leishmania* infections. As a part of our ongoing study on the phytochemical characterization and biological investigation of plants used in the traditional medicine of western and central Asia, in the present study, we focused on *Eremurus persicus* root extract in order to evaluate its potential in the treatment of leishmaniosis. As a result of our study, aloesaponol III 8-methyl ether (ASME) was isolated for the first time from *Eremurus persicus* root extract, its chemical structure elucidated by means of IR and NMR experiments and the (*R*) configuration assigned by optical activity measurements: chiroptical aspects were investigated with vibrational circular dichroism (VCD) and electronic circular dichroism (ECD) spectroscopies and DFT (density functional theory) quantum mechanical calculations. Concerning biological investigations, our results clearly proved that (*R*)-ASME inhibits *Leishmania infantum* promastigotes viability (IC_50_ 73 µg/mL), inducing morphological alterations and mitochondrial potential deregulation. Moreover, it is not toxic on macrophages at the concentration tested, thus representing a promising molecule against *Leishmania* infections.

## 1. Introduction

Leishmaniosis, a neglected tropical disease (NTD), continues to be a major health problem, affecting 12 million people worldwide [1]. This disease is caused by the *Leishmania* species, and is generally classified into three different clinical forms: visceral leishmaniosis (VL), cutaneous leishmaniosis (CL) and mucocutaneous leishmaniosis (MCL). These forms differ in the pattern and clinical manifestations of the infection. VL can be fatal if left untreated, CL is localized and frequently self-heals within 3–18 months, while MCL leaves disfiguring scars. Although new drugs, such as paromycin, miltefosine and liposomal amphotericin B, are available as antileishmanial therapy in several countries, currently pentavalent antimonials and amphotericin B are the most used drugs for such purpose. Still, they induce toxic effects, produce development of parasite resistance and, no less important, they are expensive [2]. For these reasons, the need for new chemotherapeutic drugs is still strongly felt. Among different approaches proposed to fulfill this primary need, the investigation of medicinal plants with evidence of traditional use against *Leishmania* infections represents one of the most promising strategies. In fact, several plants and plant-derived natural products have been investigated so far as antileishmanial drug candidates [3,4].

Our nature-aided drug discovery research takes place as a response to this situation. As part of our ongoing study on the phytochemical characterization and biological investigation of plants used in western central Asia traditional medicine [5], we addressed our attention to plants belonging to genus *Eremurus* (Xanthorrhoeaceae), which includes forty species mainly found in western and central Asia. In the present work, we focused on *Eremurus persicus* (Jaub & Spach) Boiss. as source of potential antiprotozoal drugs. *Eremurus persic*us has a widespread usage in Kurdistan for both food purposes and to cure diseases related to inflammation [6] and infections; it has been demonstrated that aerial parts possess antibacterial and cytotoxic properties [7] and that root extracts show anti-inflammatory properties [8]. Nonetheless, so far, no scientific evaluation of the antileishmanial properties of *Eremurus persicus* had been carried out. Therefore, we herein report the preparation of *Eremurus persicus* roots ethanolic extract, the bioassay-guided fractionation of the extract in order to identify constituents with antileishmanial potential and the isolation, structural elucidation and biological investigation of its most abundant secondary metabolite.

## 2. Results and Discussion

Maceration using ethanol is a generally employed method in selective extraction of secondary metabolites from plant tissues, and we had already utilized such a procedure in our previous works on *Eremurus Persicus* [8]. Given the long timeframes (overnight maceration) and generally low and hardly reproducible yields (3.9%–7.6%) of maceration, on the basis of our previous experience, we studied the applicability of microwave-assisted solvent extraction (MASE) to the extraction of *E. Persicus* and compared it with dynamic maceration. Pilot extraction procedures with dynamic maceration and microwave-assisted extraction were investigated keeping the ratio sample weight/solvent volume constant with a value of about 0.05 g/mL.

The maceration procedure substantially followed the one described in our previous work [8], while for MASE, the extractions were effected in closed vessel system, under controlled pressure and temperature, to minimize the losses of components due to volatilization.

Through our study, we found that a rather short extraction time (20 min) afforded the ethanolic extract (EE) in higher yields (21.2%) compared to dynamic maceration. The EE obtained from both approaches showed a superimposable chromatographic profile at HPLC-UV performed according to our previous method [9].

Following the assessment of scale-up applicability, the root ethanolic extract of *E. Persicus* was then prepared according to the optimized MASE procedure (see Section 3.3).

**Figure 1:**
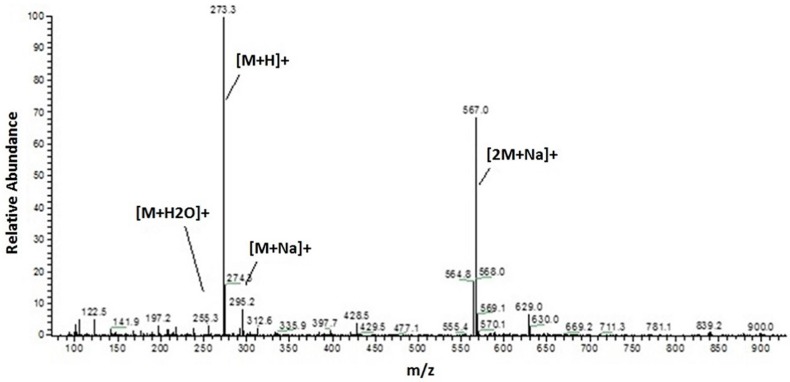
ESI-MS (positive ion mode, full scan) of the most abundant metabolite of EE.

To obtain a preliminary phytochemical fingerprint, the HPLC-UV/PAD method from [9] was exploited. The HPLC method was then slightly modified to afford faster analyses (see Section 3.4). The optimized method involves a switch in solvent from acetonitrile to methanol, which allowed to reduce chromatographic run times from 60 to 30 min without compromising the resulting information. The same method was consequently applied to both HPLC-UV/PAD and HPLC-ESI-MS analysis, with optimized MS experimental parameters for positive and negative ion modes. The HPLC-MS analysis unambiguously showed the presence of a preponderant secondary metabolite characterized by a molecular weight of 272 Da, given that the corresponding MS spectrum clearly displays an [M + H]^+^ at 273 *m*/*z*, [M + Na]^+^ at 295 *m*/*z* and a dimeric ion [2M + Na]^+^ at 567 *m*/*z* (Figure 1).

With the aim to speed up the identification of new potential antiprotozoal compounds we adopted a bioassay-guided approach. The extract was fractionated by flash chromatography on silica gel (see Section 3.5) and analyzed applying the optimized HPLC method (see Section 3.4).

**Figure 2:**
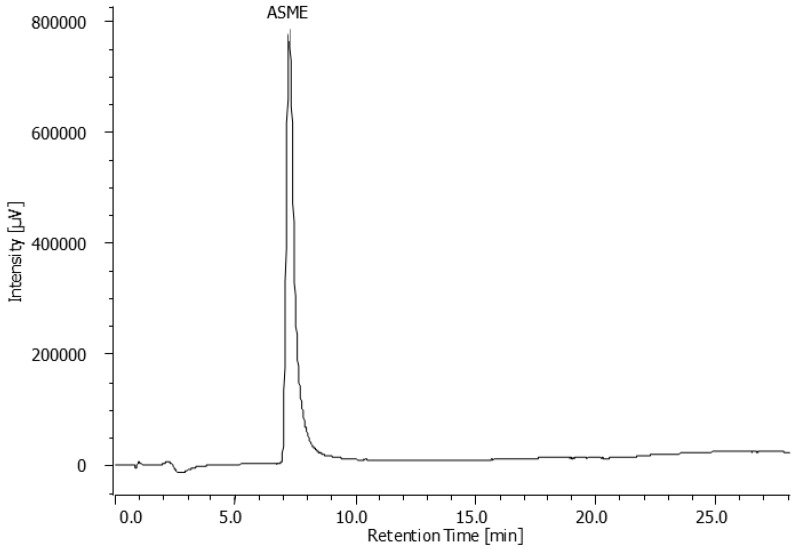
HPLC-UV chromatogram of the compound isolated through flash chromatography (λ: 270 nm).

Flash chromatography allowed the isolation of six fractions which underwent a preliminary cytotoxicity screening. Only one fraction resulted active against *Leishmania infantum* in our initial screening whose structure matches, as shown by HPLC analysis, to the most abundant metabolite (Figure 2).

In order to obtain the compound of interest in the amount and chemical purity adequate for structural characterization as well as for in depth biological in vitro investigation, liquid–liquid extraction of the crude extract using water/dichloromethane (see Section 3.6) was then experimented. This procedure allowed for the isolation of the compound of interest with higher process yields in shorter timeframes.

The structure of the isolated compound was elucidated by mono and bidimensional NMR (^1^H-NMR, ^13^C-NMR, ^1^H-^1^H COSY, ^1^H-^13^C HSQC, ^1^H-^13^C HMBC), as well as IR spectroscopy and polarimetric analysis. Overall, spectroscopic data were consistent with those reported in literature for (−)-1-oxo-4,9-dihydroxy-8-methoxy-6-methyl-1,2,3,4-tetrahydroanthracene or aloesaponol III 8-methyl ether, from now on called (−)-ASME [10,11,12].

**Figure 3:**
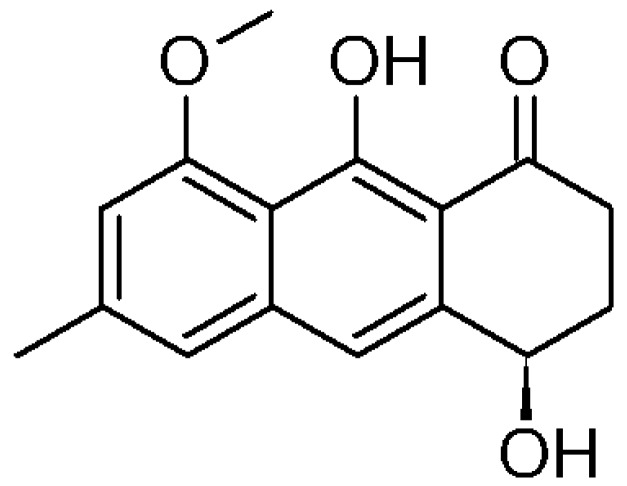
Chemical structure of (*R*)-aloesaponol III- 8 methyl ether ((*R*)-ASME).

**Figure 4:**
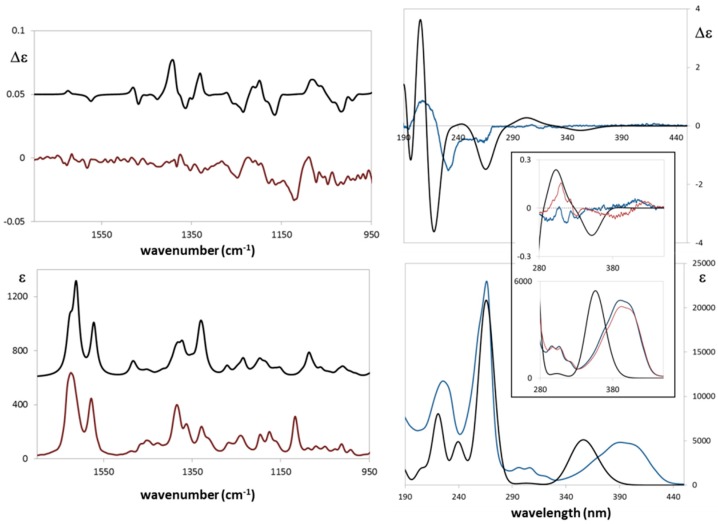
**Left**: Calculated (black trace) and experimental (red trace) VCD (top) and IR (bottom) spectra of **1**; **Right**: DFT Calculated (PCM/ACN, polarizable continuum model for acetonitrile-black trace) and experimental (blue trace) ECD (**top**) and UV (**bottom**) spectra of **1** in acetonitrile. In the inset, we repeat the magnified portions of spectra between 280 and 450 nm; in this region, we also report experimental ECD and UV data for chloroform solution (red trace). The calculations are in the PCM/CHCl_3_ approximation (see text).

The absolute configuration of (−)-ASME (Figure 3) was unambiguously assigned through VCD (vibrational circular dichroism) and ECD (electronic circular dichroism) spectroscopies and DFT (density functional theory) calculations, through the approach reported in renowned literature [13,14,15] and also already employed in instances relevant to the present case [16,17,18,19]. In Figure 4, we report the comparison of the spectra obtained with the computations performed for the assumed (*R*) configuration. The major features were correctly predicted by calculations conducted on the basis of the assumed configuration.

Even the weak features of the ECD spectra presented in the insets of Figure 4 are accounted for in the specific case of chloroform measurement: these weak features come from the subtle balance of the relative statistical weights of the ECD spectra associated to the various conformers (see Table S1 and Figure S9) and we verified experimentally that, switching to acetonitrile as solvent, a change in sign occurs as shown in Figure 4, inset. In passing, we note that, for the best prediction of most VCD and ECD data, it is crucial that the hydroxyl group on C4 be axial, as already noted in [16,17].

The minor *n*→π* feature at ca. 290 nm is crucially dependent on the population of conformers with the OH groups in the axial vs. equatorial position; in parallel, the ^1^L_b_ feature at 380 nm [20] depends on which fraction of the OCH_3_ groups is either below or above the naphthalene plane (d instead of u), as defined in Figure S9; such conformations are overall approximately 58–56% vs. 42–44% in the first case or 51–50% vs. 49–50% in the second case and might reverse with solvent change (see Table 1). The specific OR might accordingly change and we do not consider them here, since the latter data are notoriously more difficult to theoretically predict [15].

**Figure 5:**
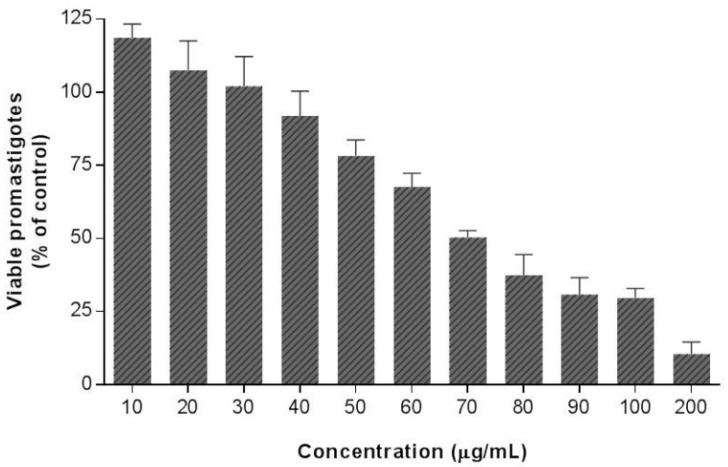
Effects of (*R*)-ASME on *L. infantum* promastigotes viability. Cultures of log-phase promastigotes (2 × 10^6^ cells·mL^−1^) were incubated at 26 °C for 24 h at different drug concentrations. Values are expressed as means and SEM.

The antileishmanial potential of EE and (*R*)-ASME was finally examined through in vitro biological investigation, in particular their antiparasitic effects were evaluated on *L. infantum* promastigotes through MTT test. The results of our study clearly suggested that EE does not possess any effect on viability of *L. infantum* promastigotes, whereas (*R*)*-*ASME showed an interesting anti-*Leishmania* activity (Figure 5), its IC_50_ value being equal to 73 µg/mL.

**Figure 6:**
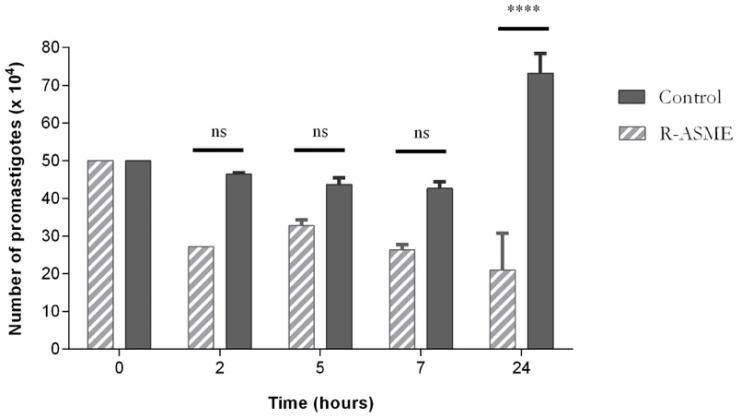
Effects of (*R*)-ASME on total cell number of *L. infantum* promastigotes, along the time of incubation. Each value represents the mean ± SEM from three independent experiments (**** *p* < 0.0001, compared to control; ns, not significant).

The treatment of *L. infantum* promastigotes with (*R*)*-*ASME at the IC_50_ concentration induced a decrease of cell number, as clearly stated in Figure 6. This effect was particularly evident after 24 h of treatment, although evidence suggests that it occurred even earlier, i.e., after five and seven hours of incubation. The decrease of cell number was probably due to the inhibition of proliferation or/and lysis of promastigotes by (*R*)-ASME.

To investigate the mechanisms of action responsible for the biological activity observed for (*R*)*-*ASME, its effects on (i) morphology; (ii) apoptosis/necrosis events; (iii) DNA integrity and (iv) cell cycle of *L. infantum* promastigotes were studied.

**Figure 7:**
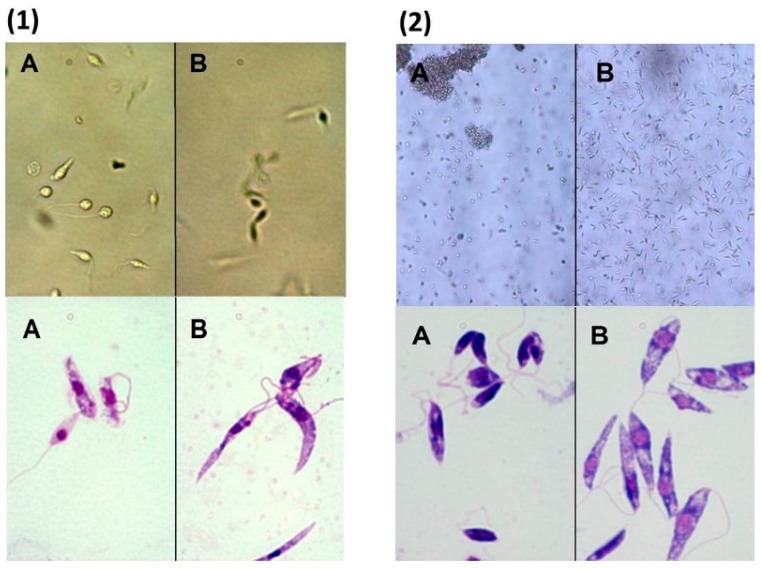
Optical microscopy observation of *L. infantum* promastigotes exposed to (*R*)-ASME (**A**); and to DMSO (control cells) (**B**); for 4 h (**1**); and 24 h (**2**). Hanging drop in phase contrast (magnification 100× and 200×) and Giemsa staining (magnification 1000×).

Concerning the effect of (*R*)*-*ASME on *L. infantum* promastigotes morphology, modifications were evaluated by optical microscopy using direct examination of live microorganism (hanging drop) and after Giemsa stain. The morphological observations pointed out a change in the shape and mobility of *L. infantum* cells treated with (*R*)*-*ASME at the IC_50_ concentration (Figure 7). In details, control cells were very mobile with characteristic fusiform shape. After Giemsa staining, it was possible to observe the characteristic shape, long flagellum emerging from the anterior region of the parasite, nucleus and kinetoplast, posterior to the nucleus. In contrast, treated cells did not show the typical elongated shape since they became rounder and smaller, losing their mobility.

This evidence suggested that cytoskeletal disorganization and/or alterations on mitochondrial bioenergetics activity occur in treated cells.

Alterations in the mitochondrial transmembrane potential were then studied by flow cytometry, using JC-1 as a probe. In healthy cells characterized by a high mitochondrial membrane potential, JC-1 tends to form aggregates; in impaired cells, with a low membrane potential, it is found as monomers. Cell treatment with (*R*)*-*ASME at IC_50_ concentration caused a loss of potential after four hours of incubation (Table 1), suggesting an early event of apoptosis mechanisms that could be associated with impaired cellular function.

To investigate the effect of (*R*)-ASME on apoptosis/necrosis events of *L. infantum* cells, the annexin-V/PI double staining method was employed. Annexin-V was used to mark early apoptotic cells, while PI, a non-permeable stain with affinity for nucleic acid, was used to distinguish necrotic cells. Our results revealed that apoptosis could be seen on treated cells after four hours of incubation and that necrosis mechanism did not occur at this time of incubation, being the percentage of cell positive to annexin-V and PI equal to 8.5 and 0.1, respectively (Table 2). After 24 h of treatment, neither annexin-V nor PI were positive, pointing out that the apoptosis/necrosis process was not maintained at this time of incubation.

Since the degradation of nuclear DNA is one of the hallmarks of apoptotic cell death, the DNA integrity of *L. infantum* promastigotes treated with (*R*)-ASME at the IC_50_ concentration was then investigated by means of gel electrophoresis DNA analysis. Interestingly, similarly to control cells, the DNA of treated cells remained intact and devoid of any fragmentation (no smears, data not shown).

Finally, the effects of (*R*)-ASME (IC_50_ concentration) on the cell cycle of *L. infantum* promastigotes were investigated by flow cytometry. (Table 3). After six hours of incubation the treated parasite cells were on G0/G1 phase (88.6%) and S phase (11%) of the cell cycle. Comparatively, in non-treated cells the number of cells on G0/G1 and S phases was 71.4% and 29%, respectively. This means that, at this time of incubation, (*R*)-ASME promoted retention of *L. infantum* promastigotes in the G0/G1 phase of the cell cycle, thus suggesting an arrest of the cycle at this stage. Interestingly, this evidence seem to be correlated with the effects of (*R*)-ASME on cell number.

Altogether, the results of our study focused on the mechanism of action of (*R*)-ASME suggest that initially (*R*)-ASME induced apoptosis events and arrest on G0/G1 phase cell cycle. Proliferation inhibition together with cell death were responsible for the rapid effects of (*R*)-ASME on cell number and morphological alterations (four to seven hours). After these events, the surviving cells are healthy; thus, at 24 h no differences in the apoptosis/necrosis parameters, DNA fragmentation and cell cycle were noticed.

**Figure 8:**
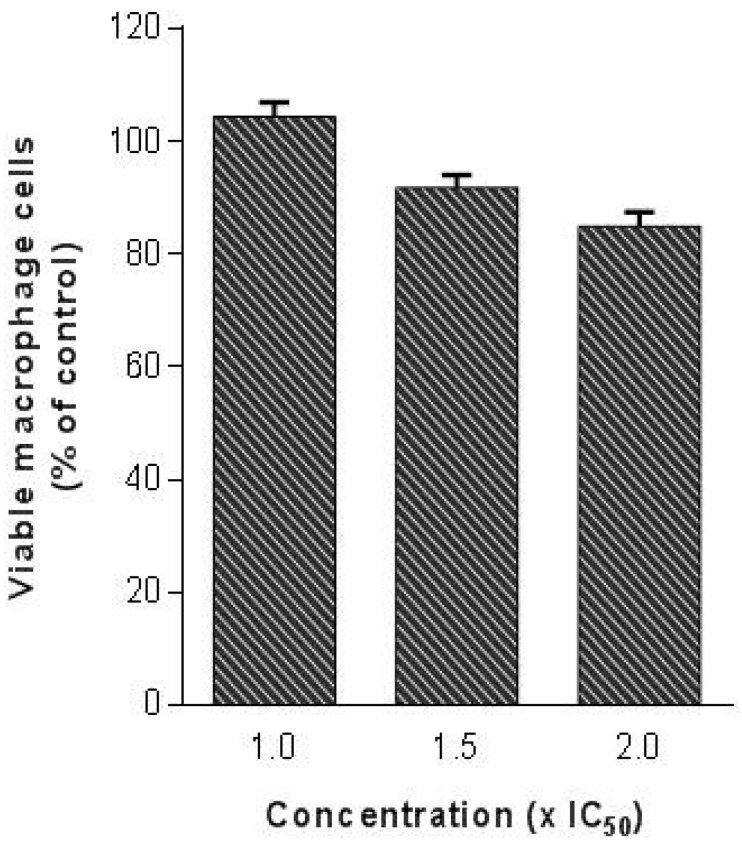
Effects of (*R*)-ASME on macrophage viability. Different concentrations (1.0 × IC_50_, 1.5 × IC_50_ and 2 × IC_50_) were tested on macrophage cells (RAW 264.7) to evaluate cytotoxicity on mammalian cells.

Since the evaluation of cytotoxicity is a highly important issue in the drug discovery process, as the last step of our study we evaluated the toxicity of (*R*)-ASME in a macrophage cell line (RAW 264.7). Indeed, it is mandatory that a molecule with anti-*Leishmania* activity does not produce significant toxicity in the host cells. In detail, (*R*)-ASME was tested at high concentrations (until two folds the IC_50_ against *L. infantum*) on mammalian cells. Results showed that (*R*)-ASME seems to be safe for mammalian cells at tested concentrations, since only a slight decrease in macrophages viability was observed (Figure 8).

## 3. Materials and Methods

### 3.1. General

Solvents for extraction, purification (analytical grade), and high performance liquid chromatography analysis—HPLC-(HPLC grade) were supplied by Carlo Erba (Milan, Italy). Polyvinylpyrrolidone (PVP), gallic acid, deuterated solvent for NMR spectroscopy, RPMI-1640 Medium, Phosphate Buffered Saline (PBS), 3-(4,5-dimethylthiazol-2-yl)-2,5-diphenyltetrazolium bromide (MTT), dimethylsulfoxide (DMSO) were purchased from Sigma Aldrich (Milan, Italy). Fetal bovine serum (FBS; GibcoR) was purchased from ThermoFisher Scientific (Lisbon, Portugal). All solvents were evaporated under reduced pressure using a Heidolph Laborota 4000 instrument (Heidolph Instruments GmbH & Co., Schwabach, Germany).

Extractions were carried out a multimode Microwave apparatus using a closed-vessel system (MARSX press, CEM Corporation, Matthews, NC, USA).

Melting points were measured on an SMP3 Stuart Scientific apparatus and are uncorrected. Analytical thin-layer chromatography (TLC) was carried out on silica gel pre-coated glass-backed plates (Fluka Kieselgel 60 F254, Merck, Darmstadt, Germany) and visualized by UV light, acidic ammonium molybdate (IV), or potassium permanganate.

Flash chromatography was performed with silica gel 60 (particle size 230–400 mesh) purchased from Nova Chimica (Cinisello Balsamo, Italy). 

^1^H-NMR, ^13^C-NMR and bidimensional experiments were performed at 400.0 MHz and 100.6 MHz, respectively, on the Bruker Avance 400 MHz FT NMR spectrometer (Bruker, Leipzig, Germany) with a multinuclear BBO probe. CDCl_3_ was used as solvent. ^1^H chemical shift values were reported on the δ scale in ppm, relative to TMS (δ = 0.0 ppm) and in ^13^C-NMR, chemical shift values were reported posing CDCl_3_ (δ = 77.36 ppm) as reference.

Optical rotation values were measured on a Jasco (Cremella, LC, Italy) photoelectric polarimeter DIP 1000 with a 1 dm cell at the sodium D line (l = 589 nm); sample concentration values (c) are given in 10^−2^ g·mL^−1^.

ECD spectra were run on a Jasco-815SE instrument (Jasco Corporation, Hachioji, Tokyo, Japan) using 0.1 to 1 mm cuvettes with chloroform and acetonitrile solutions of various concentrations (from 0.002 M to 0.004 M). VCD spectra were obtained on a Jasco FVS 6000: a 0.200 mm BaF_2_ cell and 0.056 M/CDCl_3_ solutions were employed. DFT calculations ancillary to chiroptical spectroscopies were run by use of Gaussian09 set of programs [21], preceded by Molecular Mechanics analysis of possible conformers of assumed configuration.

### 3.2. Plant Material

*Eremurus persicus* (Jaub. & Spach) Boiss. was collected in a mountain area (Kūh-e Golestān, Golpayegan) located 120 km from Isfahan/Iran, at an altitude of 3000–3200 m. The collected plant materials were identified and classified by Dr. Abdulla Sa’ad at the Education Science Department, Faculty of Biology, Salahaddin University, Hawler/Iraq. The voucher specimen (No. 6856) was deposited at ESUH (Education Salahaddin University Herbarium), Hawler/Iraq. Freshly cut roots were dried in a drying room with active ventilation at room temperature (about 20–22 °C) until they showed no further weight loss. The roots were cut to small size and grounded with a blade mill (A10 IKA-Werke GmbH & Co. Staufen, Germany) to obtain a homogeneous fine powder. The plant material so treated was stored in dark conditions.

### 3.3. Extraction Procedure

In our previous works [8,9], the ethanolic extract (EE) of *Eremurus persicus* was prepared through a dynamic maceration procedure (extraction yield 7.6%).

In the present work, the extraction process was optimized through the application of a MASE approach which gained higher yields in shorter timeframes. Briefly, the dried roots of *E. persicus* (25 g) were extracted by using ethanol (500 mL) in a multimode microwave apparatus at 120 °C for 20 min, with a power of 800 W. The extract was separated by Buchner filtration and solvent was evaporated to dryness under vacuum, yielding a yellow oil (5.2 g, extraction yield 20.8%).

### 3.4. High Performance Liquid Chromatography Analyses 

High performance liquid chromatography-photodiode array (HPLC-UV/PAD) analyses were performed on a Jasco system (Cremella, LC, Italy) equipped with a Jasco AS-2055 plus autosampler, a PU-2089 plus pump and a MD-2010 plus multi-wavelength detector. Experimental data were acquired and processed by Jasco Borwin PDA and Borwin Chromatograph Software.

High performance liquid chromatography-electrospray-tandem mass spectrometry (HPLC-ESI-MS) analyses were carried out on Finnigan LCQ fleet ion trap system, controlled by Xcalibur software 1.4 (ThermoFinnigan, San Jose, CA, USA). Mass spectra were generated both in positive and negative ion mode under constant instrumental conditions. For positive ion mode: ion spray voltage 5 kV, capillary voltage 46 V, capillary temperature 220 °C, and tube lens voltage 120 V. For negative ion mode: ion spray voltage 5 kV, capillary voltage −35 V, capillary temperature 220 °C, and tube lens voltage −100 V.

Reverse phase chromatographic analyses were carried out at room temperature (RT) under gradient conditions, using a Chromolith SpeedROD RP-18 endcapped column (50 mm × 4.6 mm, ID 3 mm, macropore size 2 μm, mesopore size 13 nm, Merck, Darmstadt, Germany). 

The HPLC analysis conditions were properly optimized to monitor the extract fractionation. The mobile phase was water containing 0.1% formic acid (A) and methanol containing 0.1%formic acid (B), and the composition gradient was: from 30% to 39% of B in 7 min, 42% B until 4 min, 50% B until 5 min followed by an isocratic elution for 2 min, 70% B until 5 min 30% B until 5 min, followed by a re-equilibration step of 2 min; total run time 30 min.

For all analyses the flow rate was set at 1 mL/min. Samples were dissolved in methanol (3 mg/mL) and filtered with a 0.45 μm GH Polypro (GHP) membrane before injection into the HPLC-system.

### 3.5. Bio-Guided Fractionation

The EE (1 g) was subjected to Flash Chromatography on Silica gel under gradient conditions eluting with 50% ethyl acetate and 50% hexane to 70% and then 100% ethyl acetate. Six fractions were collected on the basis of the TLC profile, analyzed according to described HPLC method and evaluated with a preliminary biological assay (MTT test, Section 3.8.2). Fraction 2 (19 mg, yield 1.9%), characterized by the presence of a single peak (HPLC: RT = 6.94 min, purity > 99%) corresponding to the main peak of EE chromatogram, resulted the only effective.

### 3.6. Isolation of the Main Secondary Metabolite

The EE (3 g) was dissolved in water (900 mL) and extracted with dichloromethane (DCM, 900 mL) under mechanical stirring for 3 h at RT. The organic fraction was then collected and the extraction procedure repeated for three times. The combined organic phases, dried over sodium sulphate and evaporated in vacuo furnished a yellow solid (210 mg, yield 7%, HPLC: RT 6.94 min, purity = 75%, TLC *R*_f_ 0.87 EtAc/MeOH/H_2_O). Further purification of the solid via crystallization (acetone) allowed the isolation of 135 mg of a pure compound 210 mg, m.p. 174–175 °C, yield 4.5%, HPLC: RT 6.93 min, purity > 99%). Nuclear Magnetic Resonance (^1^H- and ^13^C-NMR) and Mass Spectral (MS) techniques were employed for the structure elucidation of the isolated compound. The structure was determined as (−)-1-oxo-4,9-dihydroxy-8-methoxy-6-methyl-1,2,3,4-tetrahydroanthracene or aloesaponol III 8-methyl ether by comparison of the spectroscopic data with the previous reports.

${\left[\alpha \right]}_{D}^{20}$ −37.9 (*c* 0.04, CHCl_3_), ${\left[\alpha \right]}_{D}^{20}$ −18.2 (*c* 0.2, Acetone), ${\left[\alpha \right]}_{D}^{20}$ −18.5 (*c* 0.3, CH_3_OH); ESI-MS: *m*/*z* 273 [M + H]^+^, 295 [M + Na]^+^, 271 [M − H]^−^.

^1^H-NMR (CDCl3, 300 MHz): δ 2.01 (1H, brs, OH), 2.28 (2H, m, H-3), 2.48 (3H, s, H-11), 2.69 (1H, m, H-2), 3.05 (1H, m, H-2), 4.02 (3H, s, OCH3), 4.94 (1H, dd, *J* = 5 Hz, H-4), 6.69 (1H, d, *J* = 2 Hz, H-7 or H-5), 7.06 (1H, s, H-10), 7.14 (1H, d, *J* = 2 Hz, H-7 or H-5).

^13^C-NMR (CDCl3): δ 203.3 (C-1, s), 165.9 (C-9, s), 159.5 (C-8, s), 142.0 (C-6, s), 140.3 (C-5a, s), 139.9 (C-4a, s), 119.9 (C-5, d), 115.4 (C-10, d), 113.8 (C-8a, s), 109.2 (C-1a, s), 108.3 (C-7, d), 68.0 (C-4, d), 56.0 (OCH3, q), 34.0 (C-2, t), 30.7 (C-3, t), 22.1 (C-11, q).

### 3.7. ECD, VCD Spectra and DFT Calculations

ECD spectra were run using 0.1 to 1 mm cuvettes with chloroform and acetonitrile solutions at different concentrations (from 0.002 M to 0.004 M) to accommodate the various spectroscopic regions. Twenty scans were run to obtain data in the 500–180 nm range. The corresponding ECD spectra for the solvent in the same conditions were run and subtracted from those of the solution. VCD spectra were obtained employing a 0.200 mm BaF_2_ cell and 0.056 M/CDCl_3_ solutions. Five thousand scans were taken and solvent subtraction was made with VCD spectra in the same condition. DFT calculations ancillary to chiroptical spectroscopies were run by use of Gaussian09 set of programs [21], preceded by Molecular Mechanics analysis of possible conformers of the assumed configuration (*R*): subsequent quantum mechanical DFT calculations were conducted at B3LYP/TZVP level of theory in the PCM-IEF modelization of solvent [22]. The computed VCD and IR spectra were obtained from calculated rotational strengths and dipole strengths and wavenumbers by assigning Lorentzian bandshapes with 16 cm^−1^ bandwidth; spectra were scaled by 0.98 in frequency and divided by 3 in intensity to facilitate comparison with experimental spectra. The UV and ECD spectra were generated assigning Gaussian bandshapes with 0.2 bandwidth. Computed spectra were shifted by 20 nm and intensities were divided by 3.

### 3.8. Biological Evaluation

#### 3.8.1. Parasites and Cultures

Promastigote forms of *Leishmania infantum* Nicolle (zymodeme MON-1) were maintained at 26 °C with weekly transfers in HEPES (25 mM)-buffered RPMI 1640 medium enriched with 10% inactivated fetal bovine serum (FBS).

#### 3.8.2. Viability Assay

The antiparasitic effect of EE and (*R*)-ASME was studied through MTT test, a colorimetric assay of cell survival, following the method of [23]. Log phase *L. infantum* promastigotes were incubated with increasing concentration of EE and (*R*)-ASME, in fresh medium for 2 h. After the incubation, 25 μL of MTT (5 mg·mL^−1^) was added to each well, incubated for 2 h at 37 °C and centrifuged at 3000 rpm for 5 min. The supernatant was removed, the cells were washed in PBS, and the precipitated formazan was dissolved in DMSO (250 μL). Cell viability was measured by absorbance at 530 nm on an ELISA plate reader (Synergy HT, Bio-TEK, Winooski, VT, USA), and calculated using the following formula: [(L2/L1) × 100], where L1 is the absorbance of control cells and L2 is the absorbance of treated cells. Three separate experiments were performed for each sample and the concentration that inhibited viability by 50% (IC_50_) was determined through dose-response regression analysis, plotted by GraphPad Prism 6 (GraphPad Software, Inc., La Jolla, CA, USA).

#### 3.8.3. Morphological Studies and Cell Counting

Parasites cells were exposed to (*R*)*-*ASME and morphological alterations were investigated by optical microscopy using direct examination of live microorganisms (hanging drop) and after Giemsa stain. Briefly, exponentially grown of *L. infantum* (2 × 10^6^ cells mL^−1^) were treated with the compound at IC_50_ concentrations for 2 h, 4 h, 6 h and 24 h at 26 °C. After incubation, cells were pelleted by centrifugation at 3000 rpm for 5 min and the supernatant was discarded by aspiration. The cell pellet was suspended in fresh medium and approximately 10 μL were placed on a Koch slide and directly observed under the optical microscope phase contrast (Eclipse E400, Nikon coupled with a digital camera 165 DN100 Nikon, Nikon Europe B.V. Amsterdam, the Netherlands). In addition, a total cell counting was performed using a haemocytometer and a smear was made which was submitted to Giemsa stain. The smear was fixed with methanol for 5 min, stained with aqueous solution of Giemsa (1/10, *v*/*v*) for 10 min at room temperature and finally washed with water and air dried. The stained smear was observed under the microscope with a 100× lens (Eclipse E400, coupled with Nikon digital camera, Nikon DN100 165).

#### 3.8.4. Cell Cycle Analysis

For the analysis of DNA content, exponentially grown *L. infantum* (2 × 10^6^ cells mL^−1^) were treated with (*R*)-ASME at IC_50_ concentration for 2 h, 4 h, 6 h and 24 h at 26 °C. At each time point, cells were fixed in 200 μL of 70% ethanol for 30 min. at 4 °C. After washing cells with 2 mL of PBS, enriched with 2% of bovine serum albumin (BSA), the pellets were suspended in 0.5 mL of propidium iodide (PI) solution (PI/Rnase, Immunostep, Salamanca, Spain) and incubated for 15 min at 37 °C [24]. Cells were then analyzed by flow cytometry (D Biosciences, San Jose, CA, USA). Results were treated using ModFit LT V 2.0 programme (D Biosciences).

#### 3.8.5. Phosphatidylserine Externalization

Double staining for annexin V-FITC and PI was performed as described previously [25]. Briefly, *L. infantum* promastigotes (2 × 106 cells mL^−1^) were exposed to (*R*)*-*ASME at IC_50_ concentrations for 2 h, 4 h, 6 h, and 24 h at 26 °C. Cells were then washed with PBS and re-suspended in binding buffer (10 mM HEPES–NaOH, pH 7.4, 140 nM NaC1, 2.5 mM CaCl_2_). To 100 μL of this suspension were added 5 μL of annexin V-FITC and 5 μL of PI (AnnexinV-FITC Apoptosis Detection Kit, Immunostep). After 15 min incubation in the dark at room temperature, 400 μL of binding buffer were further added and cells were analyzed by flow cytometry (FacsCalibur–Beckton–Dickinson). Data analysis was carried out using the program Paint-a-gate, and values are expressed as a percentage of positive cells for a given marker, relatively to the number of cells analyzed.

#### 3.8.6. Measurement of Mitochondrial Membrane Potential

To assess mitochondrial membrane potential (∆ψ_m_), a cell-permeable cationic and lipophilic dye, JC-1 (5,5′,6,6′-tetrachloro-1,1′,3,3′-tetraethylbenzimidazolcarbocyanine iodide), was used as previously described [26]. This probe aggregates within mitochondria and fluoresces red (590 nm) at higher ∆ψ_m_. However, at lower ∆ψ_m_, JC-1 cannot accumulate within the mitochondria and instead remains in the cytosol as monomers, which fluoresces green (490 nm). Therefore, the ratio of red to green fluorescence gives a measure of the transmembrane electrochemical gradient. *L. infantum* promastigotes (10^6^ cells) were exposed to (*R*)-ASME at the IC_50_ concentrations for 24 h at 26 °C. Promastigotes were then incubated with JC-1 (5 µg·mL^−1^) (Molecular Probes, Invitrogen) in the dark for 15 min at RT. Then, cells were washed in PBS, suspended in 400 µL of PBS and analyzed by flow cytometry. Data analysis was carried out using the program Paint-a-gate.

#### 3.8.7. DNA Fragmentation Assay

Promastigotes of *L. infantum* (2 × 10^6^ cells mL^−1^) were exposed to (*R*)-ASME at IC_50_ concentration or to dissolution vehicle (DMSO), and incubated at 26 °C for 24 h. The *Leishmania* DNA extraction was carried out according to the procedure in DNeasy Blood & Tissue (Qiagen, Hilden, Germany). DNA integrity analysis was done by electrophoresis, running DNA through an EtBr-treated agarose gel and visualizing it with UV light.

#### 3.8.8. Mammalian Cell Cytotoxicity

For cytotoxicity assays on mammalian cells, log phase of macrophages (RAW 264.7) were trypsinized and incubated at 37 °C in 24-well tissue culture plates in Dulbecco’s Modified Eagle Medium (DMEM), enriched with Glutamax and supplemented with 10% FBS, under microaerophilic conditions. As soon as the monolayers reached confluence, the medium was removed and cells were incubated at 37 °C for 24 h with fresh medium and (*R*)-ASME at IC_50_ concentrations. After incubation, control and treated cells were washed with PBS, pH 7.2, and 450 μL of PBS and 50 μL MTT solution (5 mg·mL^−1^) were added to each well and incubated at 37 °C for 1 h. The cells were then washed with PBS, 500 μL of DMSO were added to the wells and absorbance was measured at 530 nm on an ELISA plate reader (Synergy HT, Bio-TEK). The percentage of viable cells was determined as described in the viability assay.

### 3.9. Statistical Analysis

The results are expressed as mean ± SEM from at least three independent experiments; they were analyzed by one-way analysis of variance (ANOVA), followed by Dunnett´s test, using GraphPad Prism, version 6.0d (GraphPad Software, San Diego, CA, USA). The level of significance was * *p* < 0.05, ** *p* < 0.01, *** *p* < 0.001, **** *p* < 0.0001 when compared to control.

## 4. Conclusions 

In this work, we used a bioassay-guided fractionation as a strategic approach to natural source product discovery and isolation. Liquid–liquid extraction of the ethanolic extract (EE) of *Eremurus persicus* roots combined with biological evaluation led to the identification of an active fraction with antileishmanial activity corresponding to the (*R*) enantiomer of Aloesaponol III 8-methyl ether ((*R*)-ASME). Interestingly, this is the first time that the (*R*)-ASME has been isolated from natural sources, while the (S) enantiomer had already been reported as found in *Kniphofia foliosa* [11], *Eremurus chinensis* [12] and *Asphodelus microcarpus* [27].

(*R*)-ASME possesses a remarkable antiprotozoal effect against *L. infantum*. We demonstrated that it is active with an IC_50_ of 73 µg/mL, it is able to induce alterations on both morphology and mitochondrial potential of *L. infantum* promastigotes, and does not produce significant toxicity in a macrophage cell line. Results showed that (*R*)-ASME seems to be safe for mammalian cells at tested concentrations, since only a slight decrease in macrophages viability was observed. These results strongly suggest that (*R*)-ASME may represent a valuable lead against *Leishmania* infections. Although our preliminary results are encouraging, additional studies should be planned both in vitro and in vivo to get further insight into the mechanisms of action behind the antileishmanial activity of (*R*)-ASME.

## Figures and Tables

**Table 1:** Effects of (*R*)-ASME (IC_50_ concentration) on mitochondrial transmembrane potential of *L. infantum* promastigotes.

	*L. infantum* Intracellular Entities (% of Cells)											
	JC1Ag				JC1Mon				MIF ^a^Agreg/MIFMon			
	2 h	4 h	6 h	24 h	2 h	4 h	6 h	24 h	2 h	4 h	6 h	24 h
Control	98	88	91.6	93.7	2	11.2	8.2	6.2	4	7.4	2.7	2.2
(*R*)*-*ASME	97	81.4	88.2	90.5	2.9	18.5	11.4	9.5	5.6	6	2.2	1.5

^a^ MIF: mean intensity fluorescence.

**Table 2:** Flow cytometry analysis of *L. infantum* promastigotes treated with (*R*)-ASME showing the percentage of PI and annexin-V positive cells.

	*L. infantum* Intracellular Entities (% of Cells)											
	Annexin-V				PI				Annexin/PI			
	2 h	4 h	6 h	24 h	2 h	4 h	6 h	24 h	2 h	4 h	6 h	24 h
Control	18.8	6.3	3.3	2.4	0.4	0.3	0	0.4	3.7	1.5	0.4	0.7
(*R*)-ASME	2.8	8.5	4.5	4.3	0.5	0.1	0.1	0.4	1.2	2	0.1	0.6

**Table 3:** Effects of (*R*)-ASME on the cellular cycle of *L. infantum* promastigotes.

	*L. infantum* Intracellular Entities (% of Cells)											
	Phase G0/G1				Phase S				Phase G2/M			
	2 h	4 h	6 h	24 h	2 h	4 h	6 h	24 h	2 h	4 h	6 h	24 h
Control	83.3	87.1	71.4	47.7	16	13	29	36	0.3	0	0	16.4
(*R*)-ASME	83.9	87.1	88.6	44.9	15	13	11	38	1.1	0	0	17.6

## Supplementary Materials

Supplementary materials are available online.
