# Supplementary material for: (R)-(−)-Aloesaponol III 8-Methyl Ether from Eremurus persicus: A Novel Compound against Leishmaniosis
Source: Molecules. 2017 Mar 24;22(4):519. doi: 10.3390/molecules22040519 (PMC6154379; doi:10.3390/molecules22040519)
Supplement: Supplementary file 1 [file molecules-22-00519-s001.pdf]

Supplementary Materials: (*R*)-(−)-Aloesaponol III 8-Methyl Ether from *Eremurus persicus*: A Novel Compound against Leishmaniosis

Daniela Rossi [1], Karzan Mahmood Ahmed [1,2], Raffaella Gaggeri [1,3], Serena Della Volpe [1], Lauretta Maggi [1], Giuseppe Mazzeo [4], Giovanna Longhi [4], Sergio Abbate [4], Federica Corana [5], Emanuela Martino [6,*], Marisa Machado [7,8], Raquel Varandas [9,10], Maria do Céu Sousa[9,10] and Simona Collina [1,*]

[1] Department of Drug Sciences, Medicinal Chemistry and Pharmaceutical Technology Section, University of Pavia, Viale Taramelli 12, 27100 Pavia, Italy; daniela.rossi@unipv.it (D.R.); karzchem@yahoo.com (K.M.A.); raffaella.gaggeri@irst.emr.it (R.G.); serena.dellavolpe01@universitadipavia.it (S.D.V.); lauretta.maggi@unipv.it (L.M.)

[2] Department of Science-Chemistry, University of Garmian, Kalar 46021, Kurdistan Region, Iraq

[3] Istituto Scientifico Romagnolo per lo Studio e la Cura dei Tumori (IRST) Srl—IRCCS Via Piero Maroncelli, 40, 47014 Meldola (FC), Italy

[4] Dipartimento di Medicina Molecolare e Traslazionale, Università di Brescia, Viale Europa 11, 25123 Brescia, Italy; giuseppe.mazzeo@unibs.it (G.M.); giovanna.longhi@unibs.it (G.L.); smrmachado@gmail.com (S.A.)

[5] Centro Grandi Strumenti, University of Pavia, Via Bassi 21, 27100 Pavia, Italy; federica.corana@unipv.it

[6] Department of Earth and Environmental Sciences, University of Pavia, Via S. Epifanio 14, 27100 Pavia, Italy

[7] CESPU, Instituto de Investigação e Formação Avançada em Ciências e Tecnologias da Saúde, 4585-116 Gandra PRD, Portugal; smrmachado@gmail.com (M.M.)

[8] CIBIO-UP, Centro de Investigação em Biodiversidade e Recursos Genéticos, Universidade do Porto, InBIO, 4485-661 Vairão, Portugal

[9] Faculty of Pharmacy, University of Coimbra, Pólo das Ciências da Saúde, Azinhaga de Santa Comba, 3000-548 Coimbra, Portugal; raquel.varandas@ci.uc.pt (R.V.); mcsousa@ci.uc.pt (M.d.C.S.)

[10] CNC—Center for Neurosciences and Cell Biology, University of Coimbra, Rua Larga Faculty of Medicine, Pólo I, 3004-504 Coimbra, Portugal

* Correspondences: emanuela.martino@unipv.it (E.M.); simona.collina@unipv.it (S.C.); Tel.: +39-0382-986-810 (E.M.); +39-0382-987379 (S.C.); Fax: +39-0382-422975 (E.M.); +39-0382-422975 (S.C.)

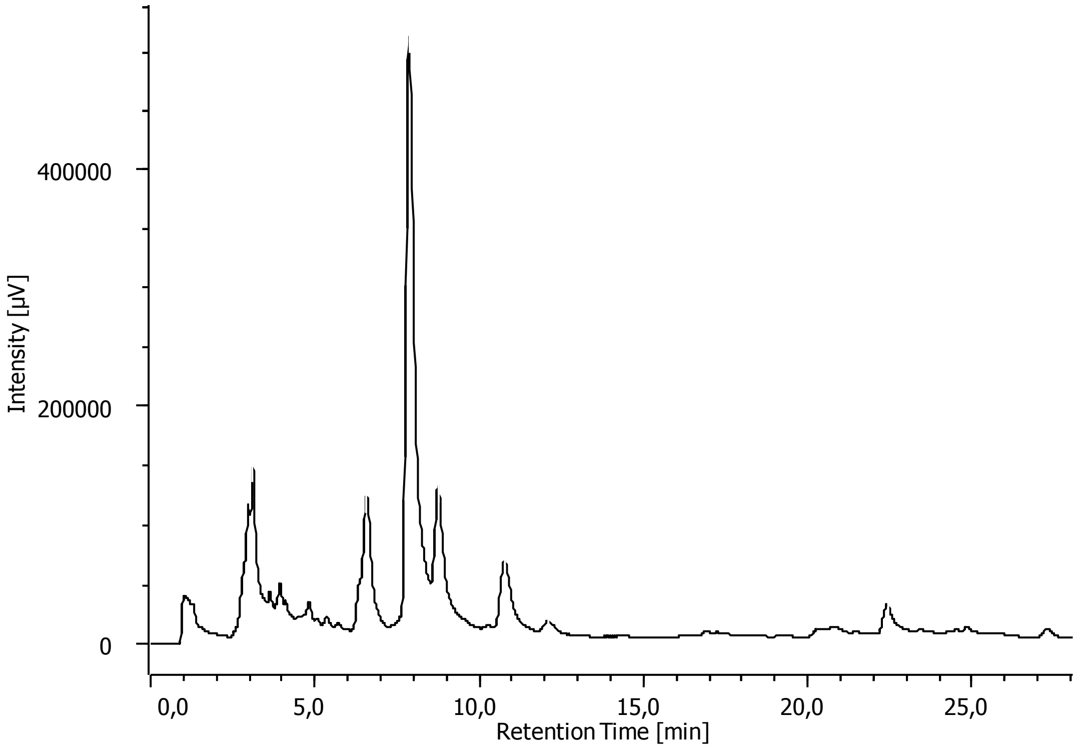

Figure S1. HPLC-UV chromatogram of MASE Ethanolic Extract, EE (λ: 270 nm).

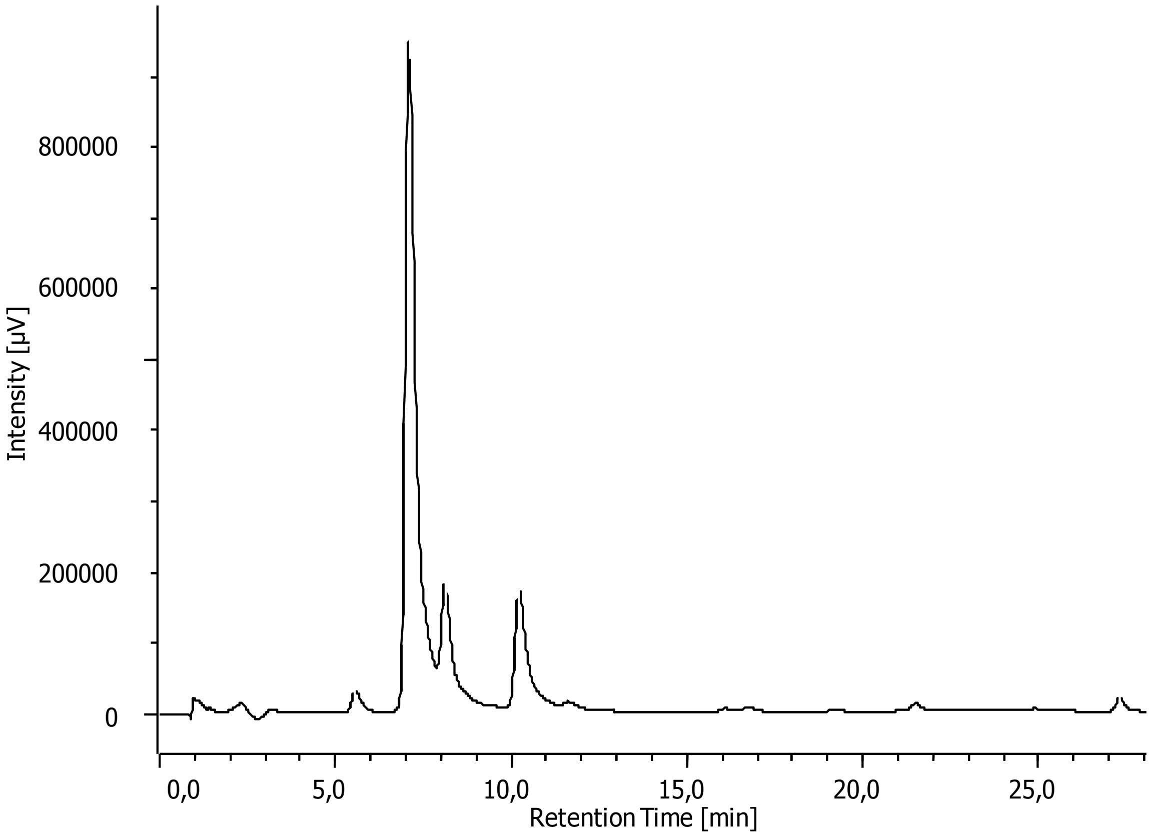

Figure S2. HPLC-UV/PAD chromatogram of the compound isolated through liquid/liquid extraction (λ: 270 nm).

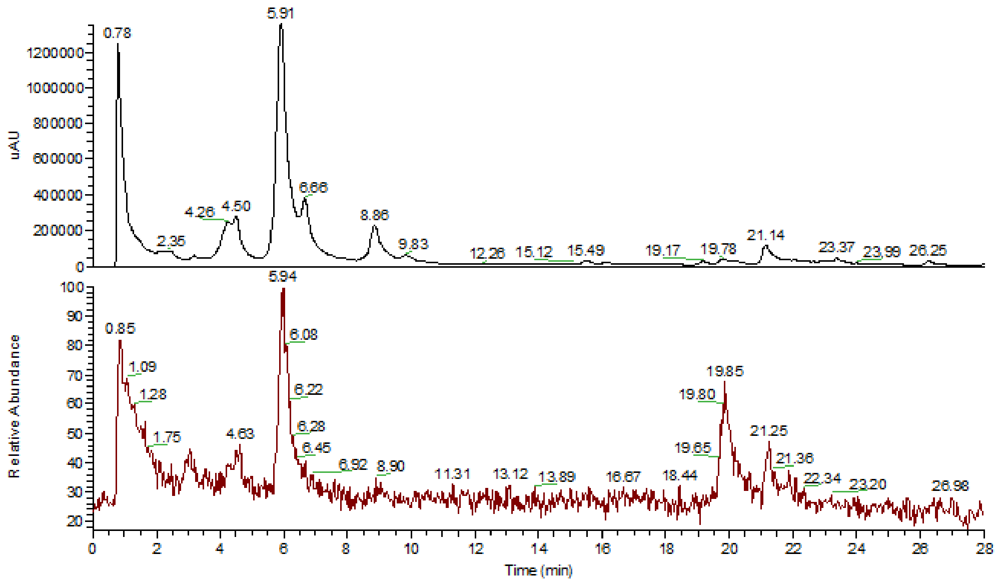

Figure S3. HPLC-UV/TIC chromatogram of MASE EE (270 nm)

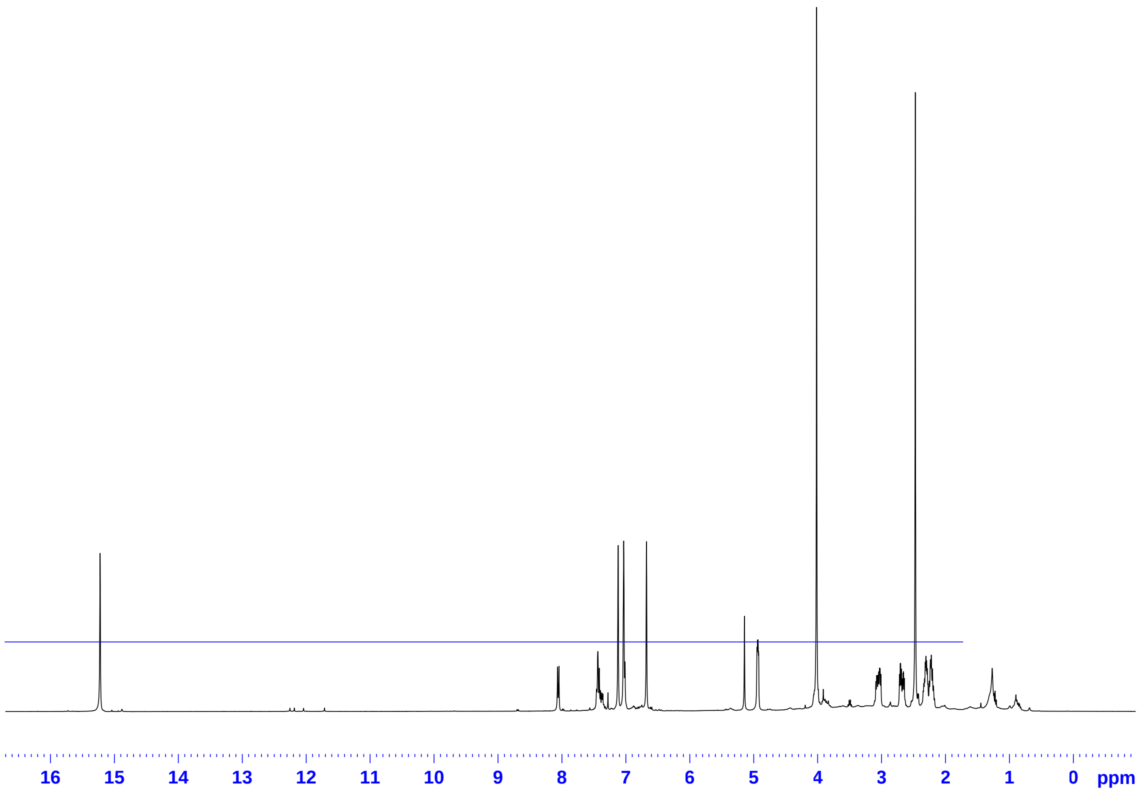

Figure S4. The proton spectrum of *(R)*-ASME.

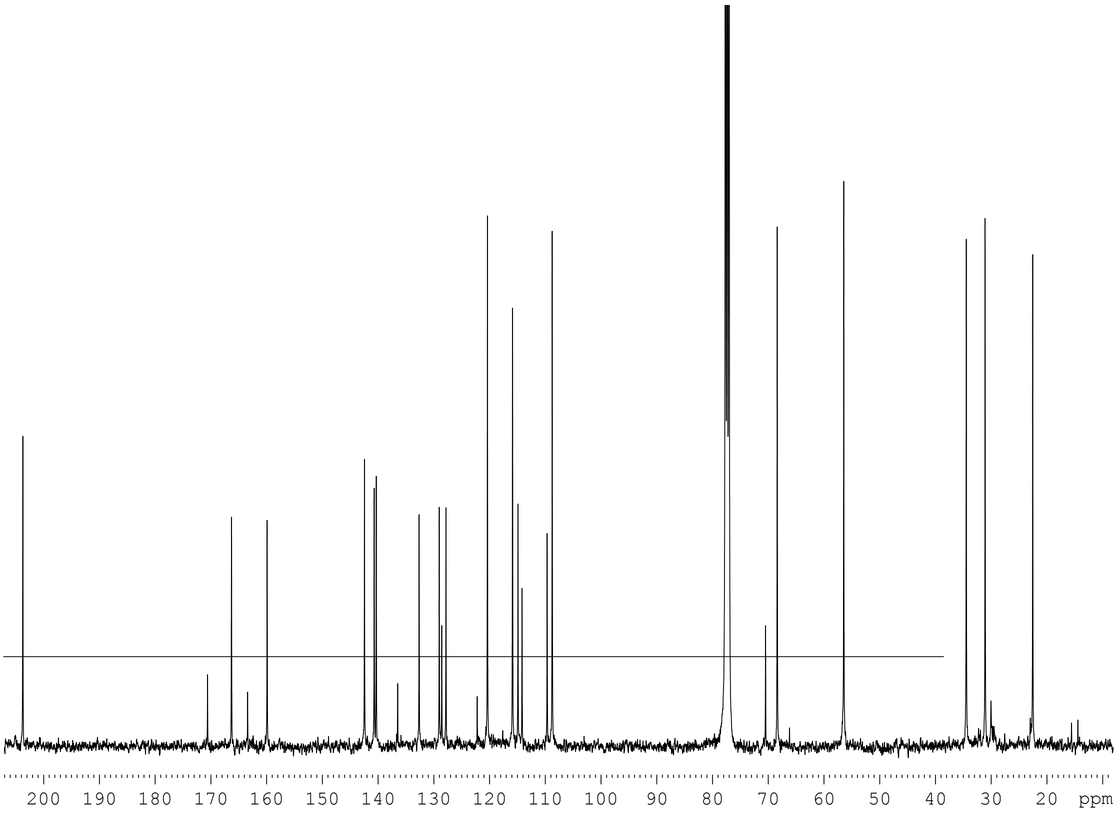

Figure S5. The carbon spectrum of *(R)*-ASME.

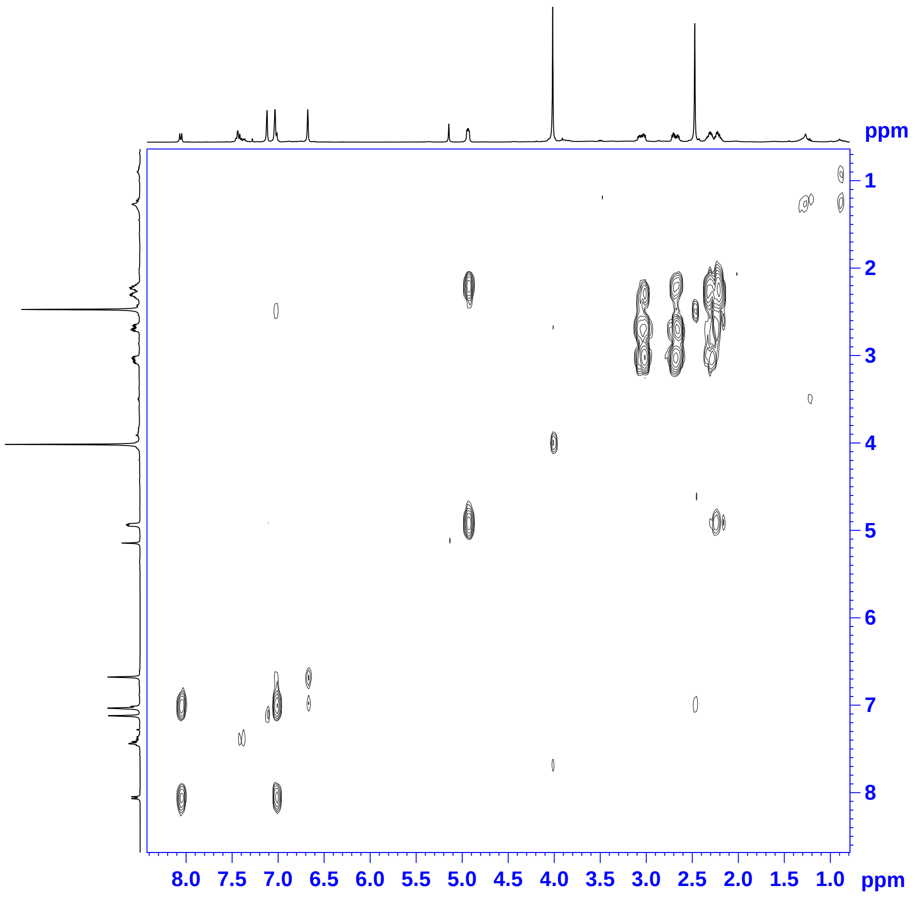

Figure S6. The COSY spectrum of *(R)*-ASME.

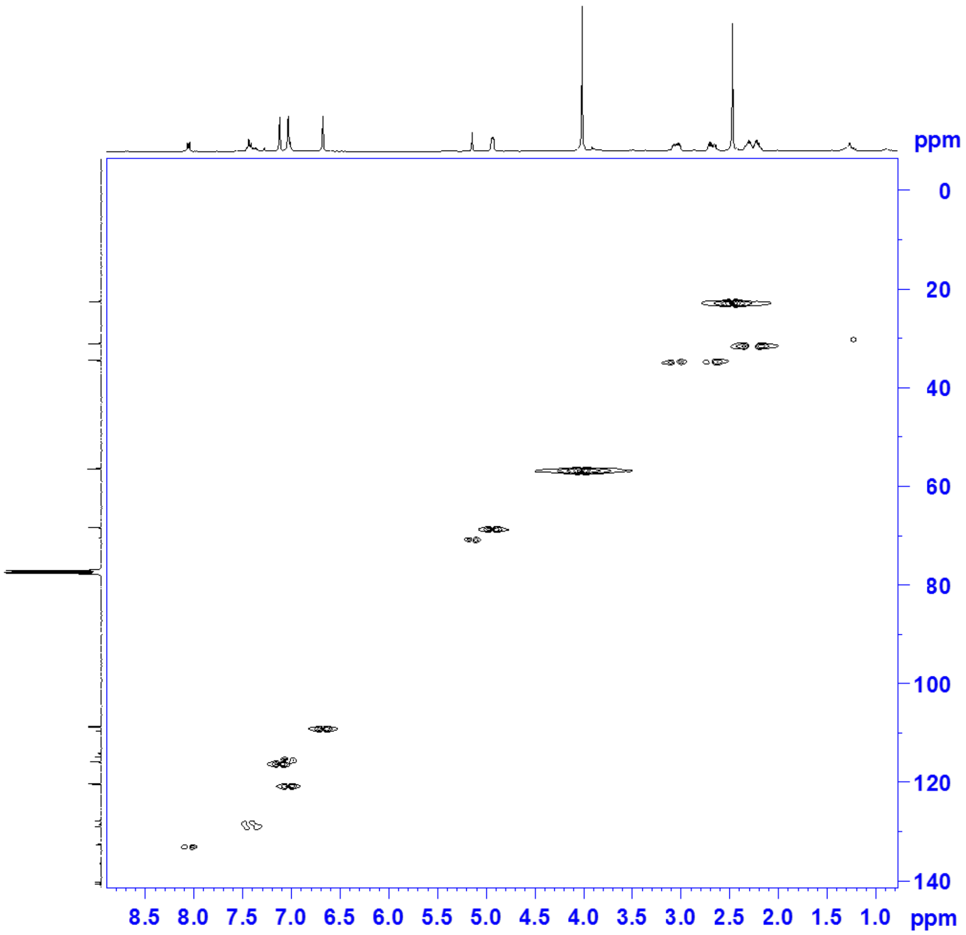

Figure S7. The HSQC spectrum capturing the short range heteronuclear correlations of *(R)*-ASME.

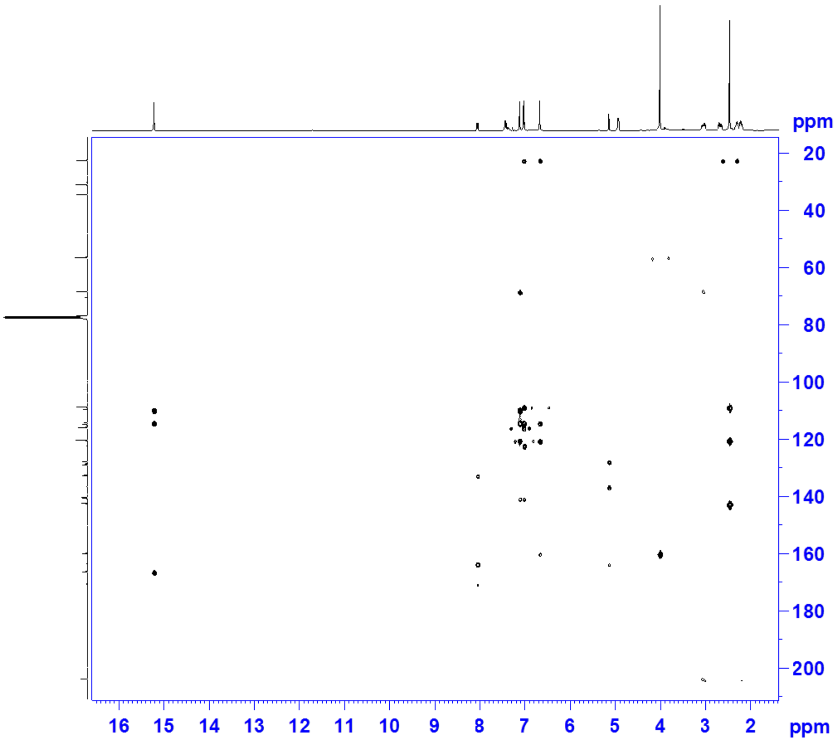

Figure S8. The HMBC spectrum capturing the long range heteronuclear correlations of *(R)*-ASME.

Table S1. Population factors for the eight most probable conformers of **1** (in order of decreasing probability). We specify the position of the non-aromatic hydroxyl group being either axial (a) or equatorial (e) and of the methoxy group being either above (u) or below (d) the aromatic plane as defined in **Figure S9**. Population factors defined proportional to $e^{-\Delta G/RT}$.

CONFORMERS	$CHCl_3$	ACN	$n \rightarrow \pi^*$	$^{1}L_b$
1(a,d)	24.0	19.4	+	+
2(a,u)	23.2	21.3	+	-
3(e,d)	14.7	13.6	-	+
4(e,u)	13.2	11.9	-	-
5(e,d)	8.0	10.5	-	+
6(e,u)	6.2	7.6	-	-
7(a,u)	6.1	9.0	+	-
8(a,d)	4.6	6.7	+	+

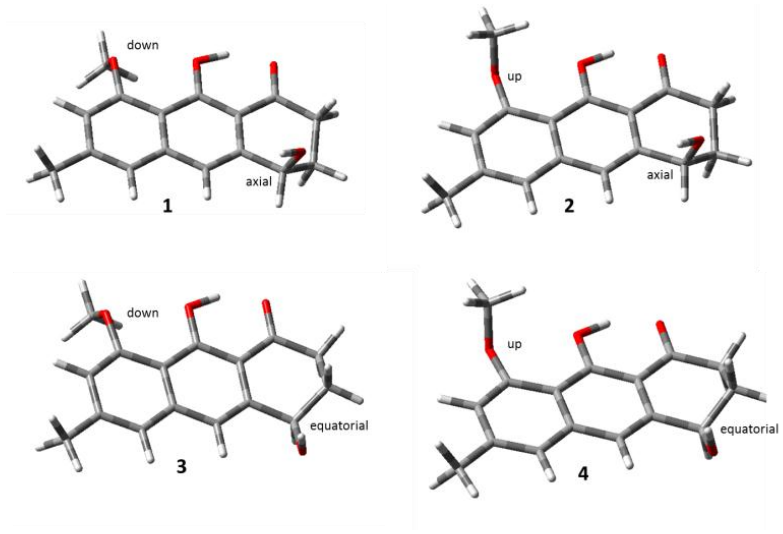

Figure S9. Representation, as calculated with Gaussian09, of the four most populated conformers of **1**: the position of the non-aromatic hydroxyl group and of the methoxy group is specified as axial (a) and equatorial (e) and as up (u) and down (d) with respect to the aromatic plane, respectively.
